# Awareness of the disease and attitude to treatment in patients with various mental disorders at the initial stages of the disease

**DOI:** 10.1192/j.eurpsy.2024.476

**Published:** 2024-08-27

**Authors:** V. Mitikhin, G. Tiumenkova, L. Alieva

**Affiliations:** Department of Mental Health Services, Mental Health Research Centre, Moscow, Russian Federation

## Abstract

**Introduction:**

An important aspect in providing effective psychiatric care and treatment is the formation of an adequate perception of their mental disorder in patients and awareness of the need for treatment, especially at the initial stages of the disease. Patients’ misunderstanding of their own psychopathological manifestations can act as a serious obstacle to their compliance and lead to an increase in the frequency of exacerbations and repeated hospitalizations.

**Objectives:**

To assess the attitude to the disease and treatment in patients with various mental disorders at the initial stages of the disease.

**Methods:**

Clinical and psychopathological, psychological, statistical. The following scales were used: Drug Attitude Inventory (DAI, Hogan T.P. et al., 1983); Insight Scale for Psychosis (ISP, Birchwood M., 1994); Questionnaire “Style of self-regulation of behavior” (SSPM, V.I. Morosanova, 1988) and others. 17 patients with a diagnosis of bipolar disorder (BD, F31.xxx, ICD-10) were examined, the average age of patients was 25.52±4.55 years and 39 patients with a diagnosis of schizophrenia (F20.1xx and F23.1xx, ICD-10), the average age of patients was 29.29±9.71. The duration of the disorder in both groups of patients was 0.5-3 years.

**Results:**

A comparative analysis of the average scores of the scale of attitude to the disease revealed significant differences in the groups (p≤0.01). Patients with schizophrenia had a lower awareness of their disease (2.31±0.91 points) than patients with bipolar disorder (3.59±0.76 points). Correlation analysis revealed reliable connections (p≤0.01) between the scales of attitude to the disease and drugs and the self-regulation questionnaire. In patients with schizophrenia, deeper violations were found in the links of self-regulation, such as programming and planning when assessing the presence of a mental disorder and deterioration of their condition due to discontinuation of medication (r=0.38 and r=0.36, respectively). The low level of self-regulation in general and the rigidity of negative attitudes in awareness of the disease and the need for treatment also have a negative impact on compliance with the medication regimen. No such correlations were found in patients with bipolar disorder: they were more aware of the presence of a mental disorder and the need for treatment, but the degree of compliance with the medication regimen was not high enough.

**Conclusions:**

The treatment of patients with mental disorders requires an integrated approach with the mandatory inclusion of a psychoeducational component in order to form an adequate model of their disease and an understanding of the expected risks when therapy is discontinued. Psychoeducation is especially relevant in the early stages of the disease, both for patients with schizophrenia and with BD.

**Disclosure of Interest:**

None Declared

